# Diagnostic Accuracy of Hysterosalpingography Compared With Laparoscopy in Assessing Tubal Patency Among Subfertile Women: A Retrospective Cohort Study

**DOI:** 10.7759/cureus.110764

**Published:** 2026-06-13

**Authors:** Swati Sharma, Ishan Wijewardana, Shilpa Deb, Taqwa Ferdous

**Affiliations:** 1 Obstetrics and Gynaecology, Queen's Medical Centre, Nottingham University Hospitals NHS Trust, Nottingham, GBR; 2 Gynaecology and Fertility, Nottingham University Hospitals NHS Trust, Nottingham, GBR; 3 Obstetrics and Gynaecology, Diana, Princess of Wales Hospital, Grimsby, GBR

**Keywords:** hysterosalpingography, infertility, screening test, sensitivity, specificity, tubal laparoscopy and dye test

## Abstract

Background

Fallopian tube pathology accounts for approximately one-third of female factor subfertility. The National Institute for Health and Care Excellence (NICE) Fertility Guidelines (CG156) recommend hysterosalpingography (HSG) as the first-line investigation for women without high-risk factors, such as pelvic inflammatory disease (PID), endometriosis, or prior pelvic surgery. However, its diagnostic accuracy varies with limitations, including tubal spasm and technical artefacts, and the contemporary data from the United Kingdom (UK) fertility services remain limited. This study evaluated the diagnostic accuracy of HSG compared with laparoscopy in detecting bilateral tubal blockage in a UK fertility clinic cohort.

Methods

We retrospectively reviewed 1,105 women aged 18-45 years who underwent HSG at NUH Life, Nottingham University Hospitals NHS Trust, UK, between January 2017 and July 2022 for subfertility (> 12 months). Exclusion criteria included BMI > 35 kg/m², incomplete records, recent PID, contrast allergy, or uncertain pregnancy status. HSG results were classified as bilateral blockage, unilateral blockage, or both tubes patent. Laparoscopy with dye testing was used as the reference standard and was offered to women with abnormal HSG tests or ongoing clinical indications. Fifty-three women underwent both procedures, of whom 49 women were included in the final analysis. Diagnostic accuracy calculations were performed using Statistical Product and Service Solutions (SPSS, version 31.0; IBM SPSS Statistics for Windows, Armonk, NY).

Results

HSG suggested tubal obstruction in 136 women (12.3%): 21 bilateral (1.9%) and 115 unilateral (10.4%). Laparoscopic correlation was available for 49 women. For bilateral tubal occlusion, HSG demonstrated a specificity of 94.3% (95% CI: 80.8%-99.3%) and sensitivity of 42.9% (95% CI: 17.7%-71.1%). For unilateral tubal occlusion, HSG demonstrated a specificity of 40.0% (95% CI: 23.9%-57.9%) and a sensitivity of 92.9% (95% CI: 66.1%-99.8%).

Conclusion

In this large cohort, HSG demonstrated bilateral tubal patency in 955 women, avoiding laparoscopy in the first instance. HSG demonstrated high sensitivity for unilateral tubal occlusion and high specificity for bilateral tubal occlusion, which supports its role as an initial screening tool. Laparoscopy remains essential for confirmation of abnormal findings and for identifying additional pelvic pathology in patients with ongoing clinical concerns. Larger prospective multicentric studies are required to provide more precise estimates of diagnostic accuracy.

## Introduction

Hysterosalpingography (HSG) is a well-established imaging modality used for the evaluation of female subfertility. It is a relatively non-invasive technique to assess the uterine cavity and tubal patency. The procedure involves an X-ray, during which contrast medium is gently passed through the cervix using a catheter to outline the uterine cavity and fallopian tubes, thereby demonstrating their morphology and the tubal patency. HSG and laparoscopy are two important investigations to evaluate tubal factors of subfertility [1]. Fallopian tube pathology is estimated to account for approximately one-third of cases of female subfertility [2]. The National Institute for Health and Care Excellence (NICE) Fertility Guidelines recommend HSG as the first-line investigation for women without high-risk factors, such as pelvic inflammatory disease (PID), endometriosis, or prior pelvic surgery [3].

However, the role of HSG is limited by substantial heterogeneity in its diagnostic accuracy, particularly when compared with the laparoscopy tubal dye test. Published comparative studies show considerable variation in the diagnostic performance of HSG, with reported sensitivity for detecting tubal blockage ranging from approximately 65% in some studies to near 100% in others, and specificity from 83% to 95% depending on study design and reference standard [4,5]. These figures support the use of HSG as an initial screening method but also highlight its limitations in reliably confirming true tubal blockage.

Several factors contribute to this variability in diagnostic performance. Technical aspects of the procedure play an instrumental role in the outcome of the results. HSG is a highly operator-dependent procedure, with experienced radiologists more likely to achieve optimal image timing and accurate interpretation. The quality of equipment used for imaging, such as the resolution of the machine and the type of contrast medium, also influences diagnostic accuracy. Oil-based contrast media have been associated with slightly higher rates of demonstrated tubal patency on HSG, likely reflecting differences in contrast properties rather than true tubal function; however, their use may be associated with an increased risk of complications such as contrast intravasation or, rarely, oil embolism [4-6]. This may explain variability in reported diagnostic performance across studies.

Transient tubal spasm during the procedure can simulate tubal obstruction, resulting in false-positive results. Similarly, previous pelvic infections, pelvic surgery, or distorted pelvic anatomy may also make the interpretation challenging. The frequency of this is hard to quantify and depends very much on the patient's variables. In addition, the timing of the study in the menstrual cycle and patient discomfort during the procedure can also affect outcomes. HSG is ideally performed in the early follicular phase to minimise the risk of pregnancy and reduce the likelihood of endometrial thickening affecting image interpretation.

Moreover, the reference standard against which HSG findings are compared significantly affects the reported sensitivity and specificity of the procedure. Studies used various reference standards, including the laparoscopic tubal dye test, sonosalpingography with saline, microbubbles (often referred to as hysterosalpingo-contrast sonography, HyCoSy), and stable gel or foam contrast agents (commonly known as hysterosalpingo-foam sonography, HyFoSy). The use of these different comparators further contributed to the heterogeneity in reported diagnostic accuracy [2,6,7].

Despite these limitations, HSG remains a commonly used first-line investigation in the evaluation of female subfertility. It provides immediate information on both the uterine cavity and fallopian tubes, as well as the pelvis. When interpreted with caution within the wider clinical context, it offers useful guidance for further management. Abnormal findings on HSG, such as suspected uterine pathology (e.g., bicornuate uterus) or tubal pathology such as bilateral tubal occlusion, should prompt diagnostic laparoscopy or hysteroscopy before initiating definitive treatment [5-9]. With the review of literature, we found that the diagnostic accuracy of HSG is understudied in the UK population. We also specifically looked at the HSG that happened during the COVID-19 pandemic period and how the delay in diagnostic tests, such as laparoscopy, affected the outcomes. In our study, we aim to evaluate the diagnostic accuracy of HSG compared with laparoscopy in detecting bilateral tubal blockage in a UK fertility cohort.

## Materials and methods

Study design and setting

This is a retrospective observational study conducted at the Fertility Centre NUH Life, Nottingham University Hospitals NHS Trust, UK. It is a diagnostic accuracy study, and follows the Standards for Reporting of Diagnostic Accuracy Studies (STARD) guidelines. It was registered as an audit to evaluate the service during the pandemic period. We retrospectively reviewed the medical records of all women who underwent HSG between January 2017 and July 2022 as part of the subfertility investigation. This represented a consecutive series of all women who underwent an HSG procedure during the study period.

Subfertility was defined as failure to conceive after 12 months of regular unprotected intercourse. The aim of our study was to evaluate the diagnostic accuracy of HSG compared with the laparoscopy tubal dye test in detecting bilateral tubal blockage. Laparoscopy is considered the gold standard for assessing tubal patency and pelvic peritoneal pathology leading to subfertility.

A total of 1,105 women who underwent HSG at our fertility unit during the study period were included by consecutive sampling. HSG was requested in accordance with institutional guidelines for assessment of female subfertility. Inclusion criteria included women aged 18-45 years undergoing HSG as an evaluation for subfertility. Women with both primary and secondary subfertility were included. Women with known tubal surgery in the past, such as salpingectomy for an ectopic pregnancy or hydrosalpinx, were not offered HSG as their preferred treatment option was in vitro fertilization (IVF). In addition, couples with significant male factor infertility, where IVF was essential, were not offered HSG.

Laparoscopy was offered and performed when there was a suspicion of tubal pathology on HSG, and based on the individual's medical history. The time interval between HSG and laparoscopy has been variable due to the influence of the pandemic, ranging between 4 and 12 months. Before HSG, baseline investigations were completed for all the patients, including pelvic ultrasound, genital-tract infection screening, mainly chlamydia, and urine pregnancy test. Exclusion criteria were acute pelvic inflammatory disease at the time of HSG, or within the preceding 12 months, BMI > 35 kg/m² (NHS funding criteria), positive pregnancy test, and hypersensitivity to the contrast medium. Exclusion criteria also include any evidence of hydrosalpinx, significant fibroid impacting the endometrium, congenital anomaly, endometrioma, or ovarian cyst on pelvic ultrasound.

All HSGs were performed according to the institutional standardised protocol to ensure consistency. There were a certain number of operators, including radiographers and radiologists, who undertake the procedure and report, respectively, who undergo competency packages within the radiology department to ensure consistency in acquiring X-rays and reporting. Nurse specialists who catheterise the uterus and inject dye undergo assessments and complete a competency package to reduce variation in practice.

On the day of the procedure, consents were confirmed, genital screening and pregnancy test were checked and confirmed negative. It was performed in a dedicated radiology X-ray room under sterile conditions. A speculum was inserted, the cervix was cleansed with sterile saline, and a sterile concentric balloon catheter (Argon Medical HSG catheter 5.5 F x 40 cm; Argon Medical Devices, Inc., Plano, TX) was introduced into the cervix. Non-ionic iodinated contrast medium (iohexol, Omnipaque 350) was instilled gradually under continuous fluoroscopy. The typical dose of dye in most of the patients is 1 mL and ranges from 1 to 5 mL. The fluoroscopy screening time is commonly around 10-30 seconds, and not more than one minute. The total procedure time is around 5-15 minutes. If tubal spasm is suspected and the patient is visibly in severe discomfort, we wait for five minutes; tubal spasm generally settles. If not, then hyoscine butyl bromide (Buscopan) 20 mg was administered intravenously. Images were obtained in anteroposterior and oblique views to evaluate uterine contour and tubal patency. A "fill-and-spill" pattern characterised by tubal opacification with free peritoneal spill was taken as evidence of patency. Bilateral tubal occlusion was diagnosed when neither tube demonstrated peritoneal spill. Unilateral occlusion was diagnosed when one tube showed free spill, and the contralateral tube showed no peritoneal spill. Following the procedure, patients were counselled regarding expected side effects and the follow-up plan. Documentation was completed on structured forms, including consent, batch numbers of all materials, and the final reporting by the consultant radiologist. Patients with indeterminate results on one side or equivocal findings were offered laparoscopy and dye test as a confirmatory diagnostic investigation. Sometimes, depending on the clinical picture, they were also given the option of assisted conception procedures, such as IVF, intrauterine insemination (IUI), or ovulation induction (OI), to help them make an informed decision regarding the laparoscopic surgery.

During the laparoscopy, methylene blue dye was used to check the tubal patency. A total of 1-2 mL of dye was diluted in 0.9% NS, and 20-80 mL was used during the procedure. Tubal patency was confirmed by visualization of dye spill from the fimbrial end. When we have seen both fill and spill, the tubes are deemed patent. When we have seen both fill and spill, the tubes are deemed patent. Bilateral occlusion was diagnosed when no dye spill was observed from either tube. There was no category allocated to the delayed spill. However, an adequate amount of up to 80 mL of dye was used to confirm that there is no fill and spill. During the laparoscopy, a 360-degree diagnostic laparoscopy is performed as standard operating protocol, and clinically significant findings such as endometriosis, adhesions, and other pathologies were systematically recorded. The collection of data is based on descriptive assessment of pathology reported on the operation note, and endometriosis is classed based on European Society of Human Reproduction and Embryology (ESHRE) guidelines.

Data collection

We retrospectively reviewed the patients' records and noted the patients' demographic information: age, BMI, and ethnicity, causes and duration of subfertility, primary or secondary subfertility, HSG findings, and corresponding laparoscopy results. Laparoscopy was performed when there was a suspicion of tubal pathology on HSG. The results were classified as bilateral blockage, unilateral blockage, or both tubes patent. IW and TF collected the data on a standard form with the listed criteria approved by the team at the beginning. SS cross-checked the results and reviewed the records as necessary, if there was any confusion with the findings.

In some patients, laparoscopy was not performed because of reasons like the patient did not consent for the procedure, or proceeded with IVF treatment, or was lost to follow up specially during COVID times when the elective services were put on hold. We excluded these cases from our calculations since we could not verify the HSG findings.

Statistical analysis

Using laparoscopy with dye as the reference standard, we evaluated sensitivity, specificity, positive predictive value, and negative predictive value with 95% confidence interval for detecting bilateral tubal occlusion.

Our primary focus was on detecting bilateral tubal blockage, as this finding most significantly impacts treatment decisions. Unilateral blockage was not analyzed as a separate outcome, as it would be difficult in a retrospective study to assess the false negatives in the group of unilateral blockage, since not every woman had a laparoscopy as a confirmatory test.

We used descriptive analysis to summarize patient characteristics and performed all analyses using Statistical Product and Service Solutions (SPSS, version 31.0; IBM SPSS Statistics for Windows, Armonk, NY).

## Results

**Figure 1:**
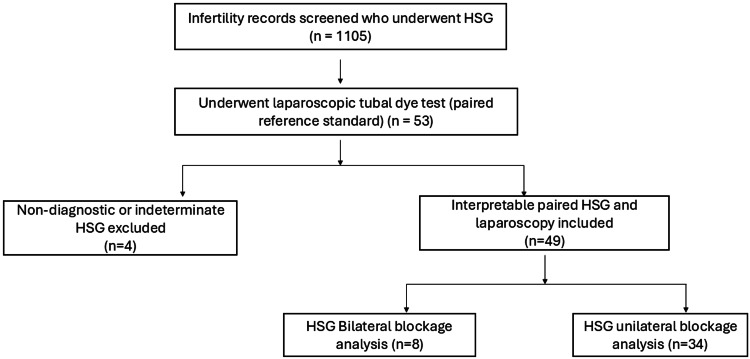
STARD flow diagram Flow diagram showing patient inclusion for diagnostic accuracy analysis of HSG compared with laparoscopy and the dye test for diagnosing bilateral and unilateral tubal blockage HSG: hysterosalpingography; STARD: Standards for Reporting of Diagnostic Accuracy Studies

A total of 1,105 patients' records were screened who underwent HSG during the study period. Among these, 53 women underwent both HSG and the laparoscopy and dye test. Four cases were excluded from diagnostic accuracy analysis because the HSG examinations were non-diagnostic or indeterminate. Therefore, 49 paired HSG-laparoscopy cases were included in the final analysis for assessment of unilateral and bilateral tubal blockage. The STARD flow diagram is shown in Figure 1.

There were no significant differences in the demographic criteria across the groups, as shown in Table 1. 

**Table 1:** Demographic criteria HSG: hysterosalpingography

HSG Group	n	Mean Age ± SD (years)		BMI	
Bilateral blockage	21	33.69 ± 5.44	p = 0.957 (Kruskal-Wallis test)	22.7 ± 2.8	p = 0.430 (Kruskal-Wallis test)
Unilateral blockage	115	32.47 ± 5.58		25.8 ± 4.1	
Bilateral patent tubes	955	33.67 ± 4.56		24.8 ± 5.9	

A total of 136 patients (12.3%) had HSG findings suggesting tubal obstruction. This included 21 women (1.9%) with bilateral tubal blockage and 115 women (10.4%) with unilateral tubal blockage.

Secondary infertility was present in 6/21 (28.6%) women with bilateral tubal blockage, as compared to 43/115 (37.4%) women with unilateral tubal blockage. There was no statistically significant difference between the type of infertility amongst the two groups (χ² = 0.60, p = 0.44).

Among the 21 women whose HSG suggested bilateral tubal blockage, eight patients underwent laparoscopy, and bilateral blockage was confirmed in six patients (28.5%). Two women (9.5%) had bilateral fill and spill on laparoscopy. Thirteen women never had laparoscopic confirmation, as one became pregnant through IVF, five declined further testing, and seven were lost to follow-up. Among the 115 women with unilateral blockage on HSG, 34 women underwent laparoscopy. Five patients (4.4%) actually had bilateral blockage of tubes on laparoscopy. Thirteen patients (11.5%) had unilateral blockage, and 16 patients (14.2%) had bilateral fill and spill. Eighty-one women did not have a laparoscopy for various reasons; 17 decided on OI, nine on IUI, and one on IVF. Out of 17 women who had OI, four had biochemical pregnancy, one woman who had IUI had twins, and the one with IVF had a live birth. Twenty-three women declined further testing, and the rest were lost to follow-up.

We were unable to perform HSG for the 12 women due to the inability to catheterise the cervix in 10 women, due to pinpoint os, or due to the inability to visualise the cervix. The procedure was abandoned in two women due to severe discomfort. Three of them had a further laparoscopy, which showed bilateral "fill and spill." There was one indeterminate result with HSG, and the woman further had a laparoscopy showing unilateral blockage. On the other hand, seven women had bilateral "fill and spill" on HSG but underwent laparoscopy due to other reasons, out of which three women had bilateral blockage, and one had unilateral blockage in laparoscopy and dye test.

The diagnostic accuracy of HSG for the detection of bilateral and unilateral tubal blockage, using the laparoscopic tubal dye test as the reference standard, is presented in Tables 2-3.

**Table 2:** Diagnostic accuracy analysis for bilateral tubal blockage cases

HSG Result	Bilateral Blockage Present on Laparoscopy	Bilateral Blockage Absent on Laparoscopy	Total
Bilateral blockage	6	2	8
No bilateral blockage	8	33	26
Total	14	35	49
Measure	Estimate	95% CI	
Sensitivity	42.9%	17.7%-71.1%	
Specificity	94.3%	80.8%-99.3%	
Positive predictive value (PPV)	75.0%	34.9%-96.8%	
Negative predictive value (NPV)	80.5%	65.1%-91.2%	
Overall accuracy	79.6%	65.7%-89.8%	

**Table 3:** Diagnostic accuracy analysis for unilateral blockage cases HSG: hysterosalpingography

HSG Result	Unilateral Blockage Present on Laparoscopy	Unilateral Blockage Absent on Laparoscopy	Total
Unilateral blockage	13	21	34
No unilateral blockage	1	14	15
Total	14	35	49
Measure	Estimate	95% CI	
Sensitivity	92.9%	66.1%-99.8%	
Specificity	40.0%	23.9%-57.9%	
Positive predictive value (PPV)	38.2%	22.2%-56.4%	
Negative predictive value (NPV)	93.3%	68.1%-99.8%	
Overall accuracy	55.1%	40.2%-69.3%	

## Discussion

This study evaluated the diagnostic accuracy of HSG for the assessment of tubal patency using laparoscopy and tubal dye test as the reference standard. HSG demonstrated high sensitivity for unilateral tubal blockage and high specificity for bilateral tubal blockage. These findings support the role of HSG as a useful first-line investigation for tubal patency while highlighting the need for cautious interpretation of abnormal findings.

The results should be interpreted in the context of the study design. It is a retrospective study, and mostly the women with abnormal HSG results were offered laparoscopy. However, in clinical practice, women with abnormal HSG findings are more likely to undergo diagnostic laparoscopy tubal dye test. Women with bilateral "fill and spill" on HSG were only offered laparoscopy if they had any associated symptoms, such as pelvic pain or ovarian cyst, to assess the gynecological condition. Similarly, some women with unilateral blockage on HSG opted directly for OI/IUI or IVF, depending on the age, cause for subfertility, and personal preferences, either to avoid surgery or to have treatment directly. Consequently, the women in the study population may have a high prevalence of suspected tubal pathology, which could influence sensitivity, specificity, and predictive values, limiting the direct applicability of the findings to other clinical settings.

Additionally, this study was conducted at a single fertility centre, and local ICB referral criteria, patient demographics, and thresholds for proceeding to laparoscopy may differ from those in other healthcare systems. Hence, caution should be exercised for the generalizability of these findings. The high specificity (94.3%) for bilateral tubal blockage and high sensitivity (92.9%) for unilateral blockage suggest that a positive finding in HSG is clinically meaningful. However, the lower sensitivity (42.9%) for bilateral blockage and low specificity (40%) for unilateral blockage indicate that HSG should not be regarded as a diagnostic test. Rather, abnormal findings should be interpreted within the broader clinical context and, where appropriate, confirmed by laparoscopy, before offering definitive treatment options. However, it can still be used as a first-line test, mainly because of its ease of performance and cost-effectiveness. Hence, these findings are broadly supportive of the NICE guidelines [3].

Our results align with some of the previous studies showing moderate sensitivity and higher specificity of HSG for tubal assessment. Gharekhanloo et al. reported sensitivity and specificity values of 98% and 96%, respectively, for bilateral tubal occlusion, which is similar to the specificity of 94.3% in our study; however, the sensitivity is much lower in our study [8]. Similarly, Khetmalas et al. showed a sensitivity of 63.64% and a specificity of 83% for HSG in detecting tubal factor for infertility [9]. This is similar to the sensitivity of bilateral tubal blockage [8]. Together, these data reinforce the view that HSG is more sensitive to bilateral tubal blockage [8-10].

Although HSG is generally safe, laparoscopy, which, while offering direct visualisation and the opportunity for simultaneous intervention, carries a higher procedural risk and anaesthetic risk. Other benefits of laparoscopy also include diagnosis and treatment of endometriosis, adhesions, and other pelvic pathology, which may be missed on HSG but are highly relevant in the context of subfertility [11-14]. Observations from our study have important implications for counselling women undergoing HSG. They should be informed that the test provides important information regarding tubal patency but is not completely accurate. Finding of bilateral tubal blockage on HSG has a high likelihood of having tubal factor infertility; however, false-positive results may occur. Conversely, a normal or unilateral tubal blockage finding does not eliminate the possibility of underlying pathology. Therefore, patients should be counselled that additional investigations, such as laparoscopy, may still be required based on the clinical situation. This is supported by published literature as well [13,14].

From a service perspective, the high specificity and reasonable sensitivity observed in this study for bilateral tubal blockage, and the high sensitivity and reasonable specificity for unilateral blockage, supported the continued use of HSG as a first-line screening tool at our centre. A planned re-audit or a prospective study following the restoration of routine surgical capacity will be important to determine whether these performance characteristics remain stable and to guide any refinement of local pathways.

The principal strengths of this study include the use of the laparoscopy tubal dye test as the reference standard and the evaluation of real-world data using a standardised HSG protocol within a fertility cohort. However, limitations include the retrospective design and the loss to follow-up. We used a longer study period to minimise loss to follow-up and to include all the women who had a laparoscopy tubal dye test at our institution by the time of data collection. However, these are still a relatively small number of paired HSG and laparoscopy cases, and there is a potential for verification bias, limiting the generalizability and precision of the reported diagnostic accuracy estimates. Notably, we were able to recommend further management for the women with normal findings on HSG, such as OI/IUI or IVF, without having to undergo laparoscopy. Only seven women underwent laparoscopy in this subgroup at our institution. However, we are not able to comment on the outcome of pregnancy in these women, as the objective of our study is only to compare the two procedures; we have not looked at the pregnancy outcome data in this subgroup.

In clinical terms, our results support the continued use of HSG as an accessible, minimally invasive first-line investigation for tubal assessment in subfertile women. However, abnormal HSG findings should prompt careful review in the context of the full clinical picture. Given the imperfect sensitivity and specificity in this study, HSG should not be considered a diagnostic test, and the laparoscopic dye test remains important for confirmation, where accurate information is needed for clinical decision making. Future prospective multicentre study designs to refine the selection criteria for laparoscopy after HSG, with all the patients followed up for a pregnancy outcome in a specific time period, might further improve diagnostic accuracy while limiting exposure to invasive procedures. Such studies should evaluate whether clinical, demographic, or imaging factors can identify women most likely to benefit from confirmatory laparoscopy following HSG [15-18].

## Conclusions

This study suggests that the HSG remains a useful first-line screening investigation for assessing tubal patency in women undergoing fertility evaluation. HSG demonstrated high sensitivity for unilateral tubal occlusion and high specificity for bilateral tubal occlusion in our study. These findings suggest that HSG can provide clinically useful information as a screening tool; however, it should be interpreted with caution and, when clinically indicated, confirmed by a diagnostic laparoscopic dye test. These findings should be interpreted in the context of the study's retrospective design, small number of paired HSG-laparoscopy results, and potential verification bias. In our opinion, within a stepwise diagnostic pathway, HSG and laparoscopy should be viewed as complementary rather than interchangeable investigations. Larger prospective multicentre studies are required to provide more precise estimates of diagnostic accuracy and to define the role of HSG in contemporary fertility evaluation and management.
